# Unraveling the Mystery: COVID-19 and Diabetic Complications - A Journey from Pathophysiology to Treatment

**DOI:** 10.2174/0115733998341202241202103725

**Published:** 2025-01-27

**Authors:** Muhammed Amanat, Sapna Tandon, Randhir Singh

**Affiliations:** 1 Department of Pharmacology, Central University of Punjab, Ghudda, Bathinda, 151401, India

**Keywords:** Diabetes mellitus, ACE2, COVID-19, SARS-CoV-2, DPP4 inhibitor, SGLT2 inhibitors

## Abstract

The connection between COVID-19 and DM unveils a multifaceted interplay that significantly impacts disease severity and management strategies. Initial studies reveal that people with DM had higher severity rates of COVID-19 due to the infection by SARS-CoV-2. The virus solely induces hyperglycemia and, at the same time, profoundly influences the immune and inflammatory reactions, increasing the rate of severe complications and death among diabetes patients. Therefore, understanding the underlying mechanisms behind this interplay is critical for effective treatment. Furthermore, COVID-19 also brings new factors to the equation of managing diabetes. Although the virus thoroughly relies on the ACE2 receptor for viral entry, DPP4 is a substitute receptor. However, glucose-lowering DPP4 inhibitors provide only a minor association with COVID-19 vulnerability. Also, the SGLT2 inhibitors are contraindicated in certain conditions with COVID-19, and hence, insulin is generally recommended as a first-line treatment for acute glycemic control in hospitalized or critically ill COVID-19 patients, particularly those with severe hyperglycemia or diabetic ketoacidosis. COVID-19-associated aggravating factors, such as cardiovascular disease, chronic kidney disease, and neuropathy, predispose people with diabetes to severe conditions. Thus, it is important to explore this speculation, and the present review aims to understand this complex interaction during patient care models and specify the therapeutic approaches to address this problematic convergence of two substantial health concerns.

## 1 INTRODUCTION

In December 2019, an acute atypical respiratory illness occurred in Wuhan, Hubei Province, China, caused by a novel coronavirus initially identified as SARS-CoV-2 and later named COVID-19 [1]. The issue persisted and became a global pandemic due to the lack of targeted therapy. On 29^th^ October 2020, WHO recorded 44,351,506 cases of COVID-19 across the globe, and the total death toll amounted to 1,171,255. Statistics on the COVID-19 fatality rate in the population: The best estimates of case fatality rates for COVID-19 range from 0.5% to 1.0% [2-4]. In America only, from March 1 to May 30, 2020, 122, 300 people died than is typical: 95, 235 of them due to COVID-19 [5]. Further, the pandemic has strained healthcare systems, overloading facilities in high-case regions and creating shortages of vital requirements like ventilators and ICU beds [6]. SARS- CoV-2 is a recently identified positive-sense single-stranded RNA virus and belongs to the Coronaviridae family of viruses, whereas 82% geo-topic identity to SARS-CoV causes SARS [7]. To infect a human cell, the virus typically uses the ACE2 receptor, which is found in lung alveolar cells, cardiac myocytes, and the vascular endothelium [8]. Human or asymptomatic transmission happens *via* respiratory droplets, and the onset of symptoms is within 5-6 days of infection [9]. Even though the infection tends to manifest minor signs at first, COVID-19 can cause severe conditions such as systemic inflammatory response syndrome, ARDS, multi-organ failure, and shock [10].

Individuals at high risk for severe COVID-19 outcomes include the elderly, males, and those with underlying conditions such as CVD, obesity, and DM (both type I and type II) [11, 12]. Early studies indicate these comorbidities are common among COVID-19 patients [13]. T2DM is more common among older people and is a risk factor for COVID-19 development, though it’s advanced age specific to T2DM is still in debate. Importantly, the respiratory system is the primary organ system affected by COVID-19. However, other organs are also affected [14]. Firstly, warning signs of COVID-19 include high body temperature, dry cough, and dyspnea, all of which are associated with lower respiratory tract infections. Later, it also included headache, dizziness, generalized weakness, vomiting, and diarrhea [15]. Moreover, immunosuppression attempts to prevent the immune system from mounting a proper defense against pathogens, which increases susceptibility to infections and immune-related challenges such as COVID-19 [16, 17]. Diabetes patients face severe COVID-19 complications due to weakened immunity, inflammation, and metabolic issues. Besides, COVID-19 can worsen glycemic control, potentially causing diabetic ketoacidosis or hyperglycemic hyperosmolar state. The cytokine storm and systemic inflammation from severe COVID-19 further aggravate insulin resistance and disrupt pancreatic beta cell function, worsening hyperglycemia in both diabetic and non-diabetic individuals [18]. Several *in vivo* and clinical studies revealed cross-talk between COVID-19 and diabetes *via* ACE2, RAGE, TMPRSS2, furin, and insulin signaling molecules (PI3K, Akt, MAPK, mTOR), impacting glucose homeostasis with significant consequences [17-20]. In the end, the pandemic devastated global health and safety, with people with diabetes being particularly affected and facing increased risks of severe illness and death from COVID-19. Hence, this review article endeavors to offer an extensive exploration of the pathophysiological mechanisms underlying diabetic complications amidst the background of COVID-19, alongside an appraisal of current treatment modalities.

## 2 CURRENT SCENARIO OF COVID-19 AND DIABETES

Diabetes is now a global issue that has grown to be of immense concern over the past few years. Currently, 536.6 million people are now dealing with diabetes in diverse regions, and this figure will rise to 783.2 million by 2045 [21]. Furthermore, studies showed that 3 out of 4 people living with the disease are in low and middle-income countries. Diabetes significantly impairs the immune system, making individuals more susceptible to infections like COVID-19. Studies have shown that diabetic patients experience prolonged recovery and more severe outcomes when infected with COVID-19 due to immune dysregulation and metabolic inflammation [22]. Additionally, animal models demonstrate that diabetes exacerbates MERS-CoV infection through altered cytokine profiles, supporting similar concerns for COVID-19 [23]. Abdominal obesity in diabetes leads to abnormal adipokine and cytokine secretion, further complicating respiratory health. Uncontrolled diabetes results in decreased interleukin production, increased asthma risk, and lower oxygen saturation, leading to mechanical respiratory issues [24]. Hypertension, however, is a common comorbidity that increases ACE2 expression, potentially facilitating viral entry and severe disease [25]. COVID-19 also disrupts hemoglobin metabolism, posing higher risks for diabetes people with elevated glycated hemoglobin [26]. In continuation, the population-based cohort study of patients with COVID-19 in England revealed that diabetes history of HbA1c > 10% were at higher risk of COVID-19 infection than patients with HbA1c < 6.5% [11].

Furthermore, reports from China indicated that there is a higher rate of hospitalization, severe pneumonia, and mortality among diabetic COVID-19 patients [27]. For instance, a case series from the USA involving 5, 700 COVID-19 patients revealed that 33.8% had diabetes. In comparison, a meta-analysis of Chinese studies showed a diabetes prevalence of 11.7% in ICU patients compared to 4.0% in non-ICU cases [28]. Another study conducted in China reported that 8.2% of 1, 590 COVID-19 patients had diabetes, rising to 34.6% among those with severe disease [29]. Consistent with this, a report from the Chinese Center for Disease Control and Prevention, covering 44, 672 COVID-19 cases, including non-hospitalized patients, revealed a lower diabetes prevalence of 5.3% [30]. Eventually, studies showed that the prevalence of diabetes among COVID-19 patients does not exceed that of the general populace. For example, in Italy, 8.9% of hospitalized COVID-19 patients had diabetes, consistent with general population rates of around 10-11% in various countries (USA, China and France) [31-33]. Nevertheless, hyperglycemia reduces immune efficacy, enhancing pathogen virulence and complicating infections. Despite not having higher infection rates, people with diabetes face more severe COVID-19 complications. The pandemic has also disrupted diabetes management, exacerbating morbidity and mortality risks for these patients. Even though Ketoacidosis is more particularly related to T1DM, it can also develop in T2DM patients during COVID-19. A systematic review showed that about 77% of the patients with COVID-19 having ketoacidosis had T2DM [34]. Therefore, optimal metabolic control and targeted therapies, such as GLP-1 agonists, are crucial in managing COVID-19 in diabetic patients [35].

## 3 STRUCTURE OF SARS-CoV-2

The structure of coronaviruses is complex and comprises various components that enable the virus to infect host cells and replicate. Coronaviruses are enveloped viruses derived from the host cell membrane during viral budding and are surrounded by a lipid bilayer membrane [36]. It contains viral glycoproteins, including the spike (S), envelope (E), and membrane (M) proteins, which are embedded in the lipid bilayer. These are crucial in protecting the viral genome, facilitating viral entry into host cells, and avoiding host immune responses. Moreover, spike (S) protein consists of two functional subunits, S1 & S2. The S1 subunit contains the receptor-binding domain (RBD) that binds to receptors like ACE2, while the S2 subunit facilitates viral and host cell membrane fusion, enabling viral entry [37]. However, envelope (E) protein is involved in viral morphogenesis, virion assembly, and pathogenesis. The membrane (M) protein is the most abundant protein in the viral envelope, and it maintains the structural integrity of the virus particle. It interacts with the nucleocapsid and envelope proteins to facilitate viral assembly [38]. In addition to aiding virus budding, the M protein interacts with host cell factors during viral entry. The nucleocapsid (N) protein binds to the viral RNA genome, forming a ribonucleoprotein complex. It is involved in viral replication, transcription, and packaging of the viral genome into new virus particles. The genome of coronaviruses consists of a single-stranded, positive-sense RNA molecule. This RNA molecule contains all the genetic information necessary for viral replication and transcription. The nucleocapsid protein encapsulates the viral RNA genome, forming the ribonucleoprotein complex, which is the template for viral replication and transcription [39, 40]. To sum up, the complex structure of coronaviruses allows them to efficiently infect host cells, replicate within them, and evade immune responses. To develop effective vaccines, therapeutics, and diagnostic tools, it is crucial to understand the structural components and molecular mechanisms of coronaviruses.

## 4 DIFFERENT VARIANTS OF SARS-CoV-2

Variants of COVID-19 are forms of the SARS-CoV-2 virus that have undergone genetic changes, leading to differences in their characteristics compared to the original strain, which emerged in late 2019. These variants can affect numerous aspects of the virus, including transmissibility, severity of illness, and the efficacy of vaccines and treatments. Though COVID-19 variants arise due to genetic mutations in the viral genome, these mutations can occur randomly during viral replication, mainly due to new variants with distinct genetic profiles [41]. COVID-19 variants are classified based on genetic differences and epidemiological significance, as described in Table **1**. These different forms are accountable for contagiousness, complexity of the sickness, or decrease in the efficacy of vaccines and treatments. Based on the literature, the main variants identified are Alpha, Beta, Gamma, Delta, and Omicron. Each variant has specific mutations in the viral genome that distinguish it from the original strain and may confer unique properties [42]. Some COVID-19 variants are more transmissible than others, which means they spread more easily from person to person. Delta and Omicron, among other variants, exhibit elevated transmissibility due to mutations in the spike protein, distinguishing them from earlier strains. Higher transmissibility can lead to a more rapid spread of the virus within communities and an increased burden on healthcare systems. Mutations in the viral spike protein, targeted by many vaccines, can affect the ability of antibodies to recognize and neutralize the virus. Some variants, such as Beta and Omicron, have been associated with reduced vaccine effectiveness, particularly against symptomatic infection and transmission [42-50].

## 5 CROSSTALK BETWEEN COVID-19 AND DIABETES: UNVEILING DIABETIC COMPLICATIONS

People with diabetes are at an increased risk of developing severe respiratory complications from COVID-19. The virus predominantly affects the respiratory system, resulting in mild respiratory distress, severe pneumonia, and ARDS. Infection occurs when the virus enters the body through inhalation of respiratory droplets containing viral particles or by touching contaminated surfaces and subsequently touching the face. Upon entry, the virus primarily targets cells in the respiratory tract, especially those that express ACE2 receptors. SARS-CoV-2 attaches to these receptors using spike proteins, allowing the virus to enter and replicate within the cells. This infection triggers an inflammatory response, causing the release of cytokines and a hyperactive immune response that damages the lung tissues and results in the development of pneumonia [17]. Eventually, the severe cases progress to ARDS, where the lungs become inflamed and filled with fluid, impairing their ability to oxygenate blood effectively, leading to respiratory failure. Complications such as fibrosis, blood clot formation, and secondary infections may further aggravate respiratory distress, posing significant challenges in the treatment and recovery of affected individuals. Also, infection with SARS-CoV-2 precipitates elevated levels of inflammation markers in the bloodstream, including lipopolysaccharides and toxic metabolites [8, 9]. However, the modulation of natural killer cell activity and IFNγ production can ameliorate these pro-inflammatory products' interstitial and vascular permeability [51-60]. Additionally, SARS-CoV-2 infection induces increased ROS production, culminating in lung fibrosis, acute lung injury, and ARDS. The augmented ROS production, alongside viral activation of the RAAS *via* upregulated angiotensin II expression, leads to insulin resistance, hyperglycemia, and vascular endothelial damage. Hence, these pathophysiological changes collectively contribute to the incidence of cardiovascular events and blood clots [17, 61-67] (Fig. **1**).

Decreased expression of ACE2, an enzyme in the epithelial cells of alveoli, small intestine, vascular endothelium, and various other human organs, is linked to DM [68]. In homeostasis, ACE2 catalyzes the cleavage of angiotensin-II and, to a lesser extent, angiotensin-I into the bioactive peptides’ angiotensin (1-7) and angiotensin (1-9). ACE2 and Ang 1-7 play a central role in the pathophysiology of COVID-19 and diabetes. In diabetic patients, ACE2 expression is dysregulated due to chronic hyperglycemia and inflammation [69]. SARS-CoV-2 binds to the ACE2 receptor *via* its spike protein, allowing viral entry into host cells, including those in the lungs, heart, and pancreas, contributing to a reduction in ACE2 expression, which further worsens insulin resistance and exacerbates hyperglycemia in diabetic individuals. Subsequently, it also leads to impaired insulin production in pancreatic β-cells, further complicating glycemic control in diabetic patients. Additionally, ACE2 has been shown to regulate the balance between pro-inflammatory and anti-inflammatory pathways by modulating the RAS. In COVID-19 patients with diabetes, ACE2 downregulation shifts this balance, leading to increased angiotensin-II activity, which drives inflammation, oxidative stress, and vasoconstriction [70]. This not only aggravates cardiovascular complications but also contributes to the onset of AKI and diabetic nephropathy in severe COVID-19 cases (Fig. **1**). However, ACE2 is downregulated as it becomes exploited by the pathogen, leaving the lungs vulnerable. Recent studies suggest SARS-CoV-2's non-structural proteins may impair hemoglobin's oxygen-carrying capacity by targeting its β1-chain [68, 71]. Additionally, there's a hypothesis that the virus may prefer binding glycated hemoglobin over non-glycated hemoglobin, though this remains speculative [71]. Moreover, DPP4 (CD26) has emerged as a potential co-receptor for SARS-CoV-2, though its role is less prominent compared to ACE2. Recent studies highlight that DPP4 affects not only glucose metabolism but also plays a role in immune regulation and inflammation [69]. In diabetic patients, elevated DPP4 activity has been linked to chronic low-grade inflammation, which may exacerbate the immune dysregulation observed in severe COVID-19 cases [69]. The interaction of SARS-CoV-2 with DPP4 could facilitate immune cells, further contributing to cytokine storms and the lethal consequences associated with COVID-19 [72]. Nonetheless, some studies have identified genetic variants that may further increase the risk of severe COVID-19 outcomes in individuals with diabetes. Polymorphisms in the ACE2 gene and related loci may modulate the expression and function of this receptor, altering susceptibility to SARS-CoV-2 [73]. Furthermore, variants in genes such as DPP4 and TLR4 are linked to altered immune responses and heightened inflammation, both of which are critical factors in the progression of severe COVID-19 complications in diabetic patients [73].

Furthermore, Guo *et al.* discovered that COVID-19 patients with diabetes have lower lymphocyte counts and higher neutrophil levels. Elevated NLR, PCT, CRP, and IL-6 levels were also observed. These inflammation markers independently contribute to severe outcomes in both diabetic and non-diabetic COVID-19 patients [74, 75]. Since the SARS-CoV-2 infection exhibits a multilayered impact on various organ systems, it exacerbates pre-existing conditions like diabetes, thereby contributing to complications like diabetic neuropathy, nephropathy, and retinopathy. Diabetic neuropathy, characterized by nerve damage and impaired neuromuscular junctions, is aggravated by the systemic inflammatory response induced by COVID-19, where the cytokine storm, marked by elevated proinflammatory cytokines, worsens nerve damage and myalgia, potentially through viral invasion of muscle tissue *via* ACE-2 receptors [76-79]. Moreover, in case of diabetic nephropathy, SARS-CoV-2 induces severe kidney injury through podocyte damage and glomerular sclerosis, mediated by cytokines like TGF-β and VEGF, leading to proteinuria and CKD [80]. The effect of the virus on kidneys is characterized by oxidative stress and mesenchymal transitions in renal cells, ultimately accelerating fibrosis and glomerular injury. However, a recent study showed that admission data for SARS-CoV-2 patients revealed heightened serum creatinine and BUN levels concomitant with a reduced GFR [81]. As a result, this renal dysfunction underscores the AKI associated with COVID-19, particularly in patients with comorbidities such as diabetes. Overall, 3.9% of these patients exhibited proteinuria, and 26.7% presented with hematuria [81]. In addition, 55% of patients were found to have AKI after the initial creatinine rise, with more than half of them moving to stage-3, as revealed by a Brazilian cohort study recently [82]. Furthermore, the potential for ocular surface infection by SARS- CoV-2 suggests a pathway for viral entry and persistence in diabetic patients, with conjunctivitis serving as an early or sole manifestation of COVID-19 [83]. In summary, the interaction of these mechanisms emphasizes the heightened vulnerability of diabetic patients to severe outcomes due to compounded viral and metabolic insults, necessitating vigilant monitoring and tailored therapeutic strategies to mitigate the amplified risk of severe diabetic complications in the context of COVID-19.

## 6 UNRAVELING THE UNDERLYING MECHANISMS: COVID-19 AND DIABETIC COMPLICATIONS

Diabetes is a persistent condition characterized by high blood sugar levels caused by a disturbance in insulin production, utilization, or both. Diabetes is also linked with numerous complications, including cardiovascular ailment, renal sickness, neuropathic pain, ocular difficulties, impaired wound healing, and susceptibility to infections. COVID-19 further complicates these by possibly increasing the severity of the illnesses and deaths. Major diabetic complications are discussed in the following.

- **Summary**

This section delves into the complex connection between COVID-19 and diabetes, focusing on how the virus exacerbates key diabetic complications, including neuropathy, nephropathy, and retinopathy. The molecular mechanisms and inflammatory processes underlying these complications are explored in detail.

### 6.1 Diabetic Neuropathy

People affected by COVID-19 have reported clinical symptoms such as nausea, headache, vomiting, dizziness, myalgia, hypogeusia, hyposmia, and unconsciousness. These collective symptoms indicate the potential involvement of the nervous system [84, 85]. Patients diagnosed with COVID-19 or those who have overcome the disease may present with several neuromuscular manifestations like facial paresis, Guillain-Barré syndrome, symmetrical neuropathy, myopathy, critical illness neuropathy, and rhabdomyolysis [86]. Most importantly, COVID-19 is associated with respiratory viruses which causes more critical symptoms in diabetic patients. Therefore, we hypothesize that diabetic neuropathy may substantially contribute to the severity of the disease. The interplay of diabetic neuropathy and dysfunction of the autonomic system may influence immune response, potentially leading to a decline in pulmonary function, especially in gas exchange. In this scenario, the nervous system intricately orchestrates innate and adaptive immunity through the inflammatory reflex mechanism. The nervous system receives sensory inputs from inflammatory cells, employing a negative feedback loop to modulate the immune response. The sensory afferent arc can be activated by humoral or neural signals, with inflammatory mediators like TNF-alpha gaining access to the central nervous scheme through circumventricular tissues. Additionally, ascending vagus fibers provide sensory innervation to immune organs, contributing afferent signals to the reflex.

The reflex motor efferent pathway causes the release of acetylcholine within the reticuloendothelial system, ultimately reducing the generation of pro-inflammatory mediators in macrophages [87]. Notably, diabetic neuropathy may assume a pivotal part in compromising the regulatory capacity of the immune response, potentially resulting in an exacerbated reaction to inflammatory stimuli. Our hypothesis suggests that dysfunction in the autonomic nervous system, affecting the balance between sympathetic and parasympathetic modulation, may contribute to the inflammatory state found in diabetic COVID-19 patients due to a disrupted inflammatory reflex. Moreover, a plausible explanation lies in the potential of diabetic neuropathy to induce functional changes in the regulation of pulmonary bronchomotor tone and ventilation control [88]. Another study noted bronchomotor tone changes in diabetic patients with accelerated I123 MIBG clearance throughout ventilatory scintigraphy, suggesting noradrenergic dysfunction in bronchial innervation [89].

### 6.2 Renal Complications

SARS-CoV-2 primarily damages the lungs but also affects other organs, especially the kidneys. Approximately 5-23% of COVID-19 patients develop AKI, categorized by elevated blood creatinine and urea, proteinuria, hematuria, and histopathological impairment. However, AKI is common in patients hospitalized with COVID-19. Recently, a systematic review and meta-analysis of 54 studies involving 30, 639 patients showed the frequency of AKI among hospitalized COVID-19 patients as 28%, with 9% requiring dialysis (Stage 3D) [90]. However, risk factors for AKI in COVID-19 include old age, male gender, obesity, diabetes mellitus, hypertension, chronic renal failure, and severe illness [91]. Still, research indicated that COVID-19 patients, along with AKI, may develop glomerular disease, as evidenced by the presence of hematuria and proteinuria [92]. The accurate mechanism remains unclear, but it was hypothesized that SARS-CoV-2 directly and indirectly impacts kidney function [93]. Moreover, SARS-CoV-2 is detected in urine samples, and the kidney damage pattern attributed to the virus appears to be localized to areas containing ACE2 receptors. The timeline of virus presence in urine aligns with the onset of AKI, suggesting a direct influence on renal function. The virus is believed to enter kidney cells by attaching to ACE2 receptors located on the membrane of glomerular podocytes and the apical membrane of proximal tubule cells. In addition to damaging kidney epithelial cells, it disturbs the equilibrium of the renin-angiotensin system [94].

Furthermore, SARS-CoV-2 infiltrates the kidneys *via* the bloodstream, leveraging extracellular vesicles for systemic dissemination. There's debate over viremia in COVID-19, but viral RNA integration into host DNA post-reverse transcription raises concerns about autoimmune responses [95]. Initial infection involves ACE2 receptor interaction and spike protein cleavage mediated by TMPRSS2 (Fig. **2**) [96]. TMPRSS2 has also been identified in the kidney, particularly within the proximal tubules [97]. Recent studies by Wang *et al.* and Chiu *et al.* have revealed that SARS-CoV-2 can enter host cells *via* the CD147-spike protein pathway, which is prominently expressed in the proximal tubules of the kidney [98]. Also, SARS-CoV-2 damages kidneys *via* inflammation, cytokine storms, coagulation activation, sepsis, hemodynamic instability, and hypoxemia [9, 98].

Consequently, recent studies implicate NRP-1 as an alternative entry route, upregulated in severe cases and notably in diabetic kidney datasets [99]. Virus entry may be responsible for podocyte dysfunction, leading to glomerular diseases such as focal segmental glomerulosclerosis (FSGS) and acute proximal tubular injury leading to tubular necrosis [100]. Of note, COVID-19 can lead to excessive IL-6 secretion, which binds to IL-6 receptors, activating vascular endothelial growth factor and reducing E-cadherin expression. This process enhances vascular permeability, potentially leading to shock and multiorgan dysfunction syndrome [101]. Moreover, in diabetic patients with kidney diseases, gluconeogenesis and glycogenolysis are diminished, making them susceptible to both hypoglycemia and hyperglycemia. Continuous RAAS activation and central metabolism disruptions contribute to proteinuria and renal fibrosis [102-104]. Recent studies have also reported instances of SARS-CoV-2 infections in people with diabetes and DKD [105].

### 6.3 Viral Penetration and Retinal Invasion

In the acute stage of COVID-19, 10% of patients had ocular symptoms, primarily affecting the ocular surface (conjunctivitis, red eye, foreign body feeling, dry eye, photophobia, fuzzy vision, itching, epiphora, ocular discomfort, and floaters). The precise mechanism is uncertain, but it was found that SARS-CoV-2 affects the ocular surface both directly and indirectly. The relationship between COVID-19 and diabetic retinopathy involves multiple interconnected mechanisms driven by the physiological impacts of SARS- CoV-2 and the broader consequences of the pandemic. The virus directly affects the retina by targeting cells expressing the ACE-2 receptor using the S subunit (S1 and S2), leading to endothelial dysfunction and microvascular damage [83]. Moreover, this is compounded by complement-mediated thrombotic microangiopathy (TMA), which contributes to microcirculatory damage [106]. Additionally, the systemic inflammation and coagulopathy associated with COVID-19 increase the risk of thromboembolic events, potentially impacting the retinal vessels. Indirectly, the pandemic has exacerbated diabetic retinopathy through lifestyle changes and reduced healthcare access. Lockdown measures have led to increased physical inactivity and poorer diabetes management, worsening insulin sensitivity and lipid metabolism, further compromising retinal health [107].

Of note, in the ocular system, RAS also plays a pivotal role in regulating fluid volume and blood pressure [108, 109]. As well, components of the ocular RAS have been identified in various parts of the eye, including the cornea, conjunctiva, sclera, ciliary body, retina, aqueous humor, vitreous humor, and iris [110]. However, in 1989, Danser *et al.* highlighted the difficulties associated with the penetration of renin-angiotensin plasma enzymes into the eye. Surprisingly, in 1996, Wagner *et al.* elucidated the local synthesis of RAS components within ocular tissues, highlighting the system's importance in regulating ocular physiology [111, 112]. Moreover, diabetes retinopathy, glaucoma, age-related macular degeneration, uveitis, cataracts, and dry eye condition have all been associated with ocular RAS [113].

Diabetic retinopathy (DR), a common but complex complication of DM, frequently leads to vision deterioration in working-age people [114]. DM animal models exhibit microvascular hemorrhage and thinning of the ganglion cells and inner plexiform layer [115]. Evidence suggests that the COVID virus elevates pro-inflammatory cytokines, such as TNF-α and IL-6, exacerbating retinal lesions due to already impaired immune response in diabetic patients [115]. Consequently, DM is a significant risk factor for poor COVID-19 prognosis, where inflammation critically aggravates retinal damage, creating a detrimental feedback loop [116]. Moreover, viral RNA of COVID-19 virus has been detected in the retinas of COVID-19 patients, suggesting that the virus can enter host cells *via* ACE2 and TMPRSS2 [117]. However, human retinal cells exhibit low ACE2 and TMPRSS2 expression in the inner nuclear layers [118, 119]. Diabetics, with their compromised innate immunity against SARS-CoV-2, allow rapid viral proliferation. High blood sugar level further weakens the immune system [120]. Moreover, DM treatments may cause overexpression of ACE2 in various organs, increasing susceptibility to SARS-CoV-2 infection [121, 122]. Another crucial factor, basigin (Transmembrane glycoprotein-CD147), has also been identified as a novel entry route for SARS-CoV-2 [123] (Fig. **3**). Unlike ACE2, CD147 is expressed in moderate-to-high levels in all human retinal cell types, especially in retinal ganglion cells. CD147 also mediates the breakdown of the neurovascular barrier induced by proinflammatory cytokines and impairs the blood-retinal barrier in streptozotocin-induced diabetic mice [124]. Therefore, in a hyperglycemic context, CD147 may facilitate SARS-CoV-2 invasion of retinal cells in people with DM.

### 6.4 Cardiovascular Complications

SARS-CoV-2 belongs to the coronavirus family, a single-stranded and encapsulated RNA virus [125] that is known to have several complications, including respiratory, cardiovascular, and neuronal diseases [126, 127]. COVID- 19 patients with diabetes and CVD face a heightened risk of severe illness and mortality [128]. Moreover, these comorbidities can worsen the outcomes of COVID-19. However, the prognosis of COVID-19 is influenced by different factors age, such as age (> 65), obesity, diabetes, hypertension, pulmonary, cardiovascular, renal, and non-alcoholic fatty liver disease [129-131].

From this perspective, cardiac injury is the most common pattern during COVID-19 [132]. A common mechanism for cardiac injury is either viral involvement of cardiomyocytes or inflammation [132]. Moreover, cardiac injury is characterized by the elevated troponin-I level [133, 134], whereas troponin-I relates to inflammation biomarkers like interleukin (IL-6), C- reactive protein (CRP), hyperferritinemia, leucocytosis [133]. Evidence reported that viral infections trigger this inflammatory activity. In the COVID case, increased ACE-2 expression on pancreatic islet cells may lead to decreased β-cell insulin reserve [135].

Furthermore, the Renin-angiotensin system (RAS) is the master regulator of ACE2. The harmony between the ACE/Ang II/AT1 and ACE2/Ang 1-7/Mas axis determines the RAS's activity. ACE-2 shows their expression either in soluble form or membrane-bound. Due to various factors like dehydration, Na^+^ deficiency, and bleeding, there is a decrease in blood volume and, subsequently, a decrease in blood pressure. Furthermore, renin is released from the juxtaglomerular cells of the kidney, resulting in the activation of RAAS system. Angiotensinogen is released from the liver and converts renin to angiotensin-I. Further, angiotensin-I converts to angiotensin-II by an enzyme, ACE [136]. Angiotensin-I shows harmful effects, including vasoconstriction, inflammation, fibrosis, cellular growth and migration, as well as fluid retention. ACE-2 enzyme converts Angiotensin-II to angiotensin 1-7, while its mass receptor constitutes the protective arm of the RAAS and shows beneficial effects, including vasodilation, inhibition of cell growth, anti-thrombotic, and anti-arrhythmogenic actions [137]. Therefore, inhibition of ACE-2 causes more accumulation of angiotensin-II, resulting in inflammation, vasoconstriction, and fibrosis, decreasing insulin production from beta cells.

Spike glycoprotein of SARS-CoV-2 binds to ACE-2, resulting in downregulation of ACE-2 and proteolytic cleavage of ACE-2. Different variants of spike glycoproteins (alpha, beta, gamma, and delta) have been found in the literature, whereas delta has more affinity towards ACE-2 and blocks SARS-CoV-2 binding [138]. In addition, mutation of delta protein causes downregulation of ACE-2. In pancreatic beta cells, NRP-1 binds to the furin-cleaved substrate of SARS-CoV-2, which regulates the expression of ACE-2 & viral entry [99]. In the necroptotic cell, HMGB1 is a DNA-binding protein that regulates chromatin and the expression of ACE-2 & viral entry. Furthermore, before the entry of SARS-CoV-2, two proteolytic processing steps occur, which are mediated by host cell two proteases such as furin and cathepsin L, which are responsible for fusion with the membrane of the target cell [96]. The entry of SARS-CoV-2 has strong binding affinity towards ACE-2 and facilitates the viral entry into the target cell by transmembrane proteases serine 2 (TMPRSS2) [139]. ACE-2 binds to S-protein is known as S-protein priming, resulting in downregulation of ACE-2 and upregulation of SGLT-1 due to lysosomal degradation. Upregulation of SGLT-2 forms precipitate and causes hyperglycemia [140]. Therefore, a protective arm of RAAS removes & activates microphage, which produces cytokines (cytokine storm) [137] by regulating disintegrin and metalloproteinase 17 (ADAM17); also responsible for proteolytic cleavage of ACE-2, which causes cytokine-induced hyperinflammation (Fig. **4**). In addition, CD14^+^ CD16^+^ monocytes elevate the level of IL-6, prolong the cytokine storm, and lead to inflammation [141] elevation in ACE-2 expression, susceptibility to COVID increases. Eventually, due to elevation in D-Dimer level, hypercoagulability and pulmonary embolism occur, which causes hypoxia and, ultimately, heart failure. However, various mechanistic pathways include direct myocardial injury, systemic inflammation, electrolyte imbalance, altered myocardial demand also responsible for myocardial inflammation and myocardial fibrosis (Table **2**). Due to proteolytic cleavage of ACE-2, direct myocardial injury occurs. Myocardial supply and demand ratio altered, leading to hypoxia and further acute respiratory illness and acute myocardial injury [142]. Myocardial inflammation results in a decline in the strength of cardiac contractility, elevates filling pressures and generates ionotropic deficits, leading to acute heart failure. Endothelium dysfunction impairs the synthesis of vasodilators and increases procoagulant and vasoconstriction—impairment of cardiac remodelling due to myocardial fibrosis [9].

## 7 TREATMENT APPROACHES

The treatment approach for patients with COVID-19 and DM requires careful consideration of various factors, including the severity of COVID-19, the specific antidiabetic medications being used, and the overall health and comorbidities of the patient.

- **Summary**

This section outlines therapeutic strategies for managing diabetic patients with COVID-19, focusing on maintaining glycemic control and adjusting treatments in light of the disease severity. Moreover, this evaluates the roles of insulin, DPP4 inhibitors, GLP-1 analogues, and other antidiabetic agents in balancing the dual challenge of diabetes and COVID-19.

Patients with diabetes are at an increased risk of severe illness from COVID-19, primarily due to the interplay between hyperglycemia and the immune response. Poorly managed diabetes can lead to a compromised immune system, characterized by reduced lymphocyte proliferation, altered phagocyte response, and abnormalities in hypersensitivity and complement protein activation. High blood glucose levels have been shown to promote viral replication and worsen pulmonary function, further complicating the clinical picture for diabetic patients with COVID-19 [143-148].

### 7.1 Monitoring and Initial Management

Regular monitoring of blood sugar levels is critical in diabetic patients with COVID-19, both during hospitalization and in outpatient settings [149]. Mild infections should commonly be managed with standard diabetes care, avoiding medications like SGLT2 inhibitors that can increase the risk of dehydration and diabetic ketoacidosis. Metformin, commonly used for its anti-inflammatory properties, should be discontinued in cases of moderate to severe illness due to the risk of lactic acidosis [150]. Moreover, adjustments to oral hypoglycemic agents like sulfonylureas should be made based on blood glucose levels, dietary intake, and the risk of hypoglycemia, with a strong recommendation for discontinuation in hospitalized patients [151].

### 7.2 Choice of Antidiabetic Medications

#### 7.2.1 Insulin Therapy

For hospitalized patients, particularly those who are critically ill, insulin remains the preferred treatment for T1DM. Insulin infusion significantly reduced inflammatory markers like IL-6 and D-dimer, which are often elevated in severe COVID-19 [152]. Fast-acting insulin is used to manage acute hyperglycemia, with continuous monitoring to adjust dosages as needed [153]. Moreover, insulin therapy must be sustained in all hospitalized COVID-19 patients with T2DM who were on insulin before admission, as maintaining stable basal insulin levels to prevent severe hyperglycemic crises [154]. Patients who typically use more concentrated insulin, like U-500 regular insulin (500 units/ml), can continue this regimen in the hospital if available. Due to the high concentration of U-500 insulin delaying absorption, its pharmacokinetic profile is similar to that of NPH insulin. Consequently, if U-500 insulin is unavailable, U-100 NPH is a suitable alternative [155]. However, clear communication among healthcare providers and patients is essential to ensure proper dosing and avoid errors, especially during the COVID-19 pandemic, where staffing shortages may pose additional challenges.

#### 7.2.2 DPP4 Inhibitors and GLP-1 Analogues

These medications are recommended for patients with mild to moderate COVID-19 symptoms and have proven efficacy in lowering glucose levels and possess anti-inflammatory properties, making them suitable for less severe cases. So far, ACE2 is the primary receptor for SARS-CoV-2, but DPP4 may also bind to the virus [156]. Surprisingly, some DPP4 protein variants in Africans are linked to a lower risk of MERS-CoV infection [157, 158]. However, patients with MERS-CoV showed significantly lower plasma levels of DPP4, suggesting DPP4 may have a protective role [158]. Still, their use in critically ill patients is less supported due to insufficient data. Moreover, a study showed systemic DPP4 inhibition increased mouse inflammatory markers [159]. However, in humans, DPP4 inhibitor (DPP4i) treatment decreased DPP4 enzyme activity without raising inflammatory markers [159], thereby suggesting a potential difference between animals and humans regarding DPP4 activity and inflammation. Additionally, DPP4i treatment suppressed T-cell immune responses to the COVID-19 virus in human blood samples [160]. Another human study reported decreased brain-derived neurotrophic factor levels with DPP4 inhibition [161]. So, the impact of DPP4 inhibition on T cell function and immune responses in COVID-19 patients needs further investigation.

In patients with T1DM, infusions of GLP1 decrease plasma levels of IL-6, ICAM 1, and oxidative stress markers [162]. GLP1 and its analogs have shown benefits in treating chronic inflammatory diseases such as NAFD, atherosclerosis, and neurodegenerative disorders by reducing inflammation [163, 164]. Although it's unclear if these anti-inflammatory effects are relevant for COVID-19, there is no significant concern about continuing GLP1 analog use in diabetic patients with COVID-19. As well, patients with CVD or renal complications have a worse prognosis with COVID-19, making it essential to maintain cardiorenal health [165]. Also, it was established that GLP1 analogs exhibit good results in preventing CVD and kidney disease, making these drugs ideal for treating diabetic patients at high risk [166, 167]. Besides, overweight and obesity present significant challenges during the COVID-19 pandemic. Patients with these conditions often have compromised immune function, which can exacerbate the severity of COVID-19 [168]. Moreover, COVID-19 patients with obesity have shown poorer lung compliance and worse outcomes. Thus, GLP1 analogs, which aid in weight reduction, are recommended for obese patients with T2DM [169]. However, continuing these treatments during severe COVID-19 is not advisable due to the slow titration needed and potential side effects like nausea and vomiting provoke [170].

#### 7.2.3 Metformin

While metformin has anti-inflammatory benefits and has been associated with lower in-hospital mortality rates, its use should be conservative, particularly in patients with respiratory issues [171]. It is recommended for patients with mild COVID-19 who are not critically ill [68].

#### 7.2.4 Sulfonylureas and Thiazolidinediones

In the context of COVID-19, Sulfonylureas can cause hypoglycemia and are not recommended for severe cases [172], while thiazolidinediones can lead to weight gain and edema, potentially aggravating heart failure and thus are not recommended for severe COVID-19 [68]. Nevertheless, the ongoing administration of sulfonylureas, particularly short- acting ones like gliclazide, can be justified for stable hospitalized COVID-19 patients who can maintain a regular diet. However, unforeseen changes in meal intake, especially in ICU settings, could elevate the risk of severe hypoglycemia. As we know, COVID-19 can cause cardiac injury; precaution is suggested when using sulfonylureas in patients with severe COVID-19 during hospitalization. In addition, sulfonylureas regarding MI patients are considered a cautious drug due to their non-selective binding affinity with both cardiac and pancreatic receptors. Therefore, newer sulfonylureas like gliclazide and glimepiride, which selectively bind to pancreatic receptors, may be considered safer than older non-selective medicines [154].

#### 7.2.5 SGLT2 Inhibitors

In T2DM, SGLT2 acts on the kidneys to lower blood glucose levels. Based on the evidence, SGLT2 has been found to induce a mitigating effect on the inflammatory response allied with arterial plaque formation [173]. Moreover, they modulate mRNA expression levels about certain crucial cytokines and chemokines within the inflammatory cascade, notably TNF-α, IL-6, and MCP1 [174]. Still, due to the risk of dehydration and DKA, SGLT2 inhibitors are not recommended in critically COVID-19 patients [175]. Of note, an ongoing study, registered as NCT04350593, is actively investigating the comparative impact of dapagliflozin versus a placebo regimen. Administered once daily for 30 days, this trial aims to clarify the potential of dapagliflozin in mitigating disease progression, curtailing complications, and ultimately reducing all-cause mortality among patients hospitalized with COVID-19 [176].

### 7.3 Managing Complications

So far, improper management of diabetes or prolonged diabetes leads to diabetic complications. Hence, maintaining blood glucose levels is crucial for preventing these complications. Moreover, the SARS-CoV-2 virus further complicates the situation for diabetic patients. For DKA, specific guidelines emphasize using subcutaneous insulin regimens and frequent monitoring of blood glucose and ketone bodies. Proper management of fluids and electrolytes is crucial, particularly in patients with impaired respiratory function [154]. General recommendations for fluid management should be followed, with no specific modifications for diabetic patients beyond standard care. However, maintaining glycemic control in ICU patients with COVID-19 is challenging due to high insulin requirements and potential glucose fluctuations caused by intravenous steroids, vitamin C, and other medications. A tailored insulin regimen should be devised based on individual needs and continuously adjusted [154]. Besides that, Anakinra, an interleukin-1 receptor antagonist, is used in severe COVID-19 patients with secondary hemophagocytic lymphohistiocytosis to modulate the immune response [177]. It has also shown promising results by decreasing hyperinflammation and the cytokine storm, making it a potential treatment option for severely ill COVID-19 patients with acute respiratory distress syndrome (ARDS) [178]. Of note, COVID-19 presents another complication for diabetic patients by exacerbating their underlying immune system dysfunction. This heightened state of immunocompromise renders diabetics particularly vulnerable to severe complications from the virus, necessitating meticulous management of both their glycemic control and the hyperinflammatory response induced by COVID-19. In this context, Baricitinib, a JAK inhibitor, has effectively addressed immune dysregulation in severe COVID-19 cases [179]. By inhibiting the Janus kinase pathways, Baricitinib effectively modulates the hyperinflammatory response frequently observed in critically ill patients, thereby attenuating the severity of immune-mediated damage and offering a promising therapeutic avenue for this high-risk population [132, 180].

While acute glycemic control is essential to mitigate immediate complications in diabetic patients with COVID-19, long-term management remains critical for preventing chronic complications and ensuring better recovery outcomes. The post-COVID-19 phase presents unique challenges for diabetic patients, as persistent inflammation, immune dysfunction, and potential insulin resistance contribute to the worsening of diabetic conditions. Studies suggest that long-term follow-up should include regular monitoring of blood glucose, kidney function, and cardiovascular health to identify early signs of chronic complications. Furthermore, pharmacological interventions such as SGLT2 inhibitors and GLP-1 receptor agonists offer additional benefits beyond glycemic control. These drugs have anti-inflammatory and cardio-protective effects, making them ideal candidates for long-term management in post-COVID-19 diabetic patients [181-185]. Continuous lifestyle interventions, such as dietary modifications and exercise, also play an essential role in improving insulin sensitivity and reducing the risk of long-term complications. We have summarized different treatments for diabetic complications in Table **3**. In summary, it can be concluded that the treatment approach for diabetic patients with COVID-19 involves a nuanced strategy that balances effective glycemic control with managing COVID-19 symptoms and complications. The choice of antidiabetic medications should be guided by the severity of the illness, with insulin being the preferred option for critically ill patients. Regular monitoring, careful management of complications, and a multidisciplinary approach are essential to optimize outcomes for these patients [186-200].

## 8 FUTURE PERSPECTIVES

In December 2019, coronavirus (COVID-19) infection was considered a worldwide pandemic. After COVID-19 infection, symptoms like fever, dry cough, and shortness of breath can progress to severe respiratory failure. Most COVID patients were found to have pre-existing conditions like diabetes, asthma, or hypertension. The WHO report highlighted that individuals with concurrent diabetes and hypertension were particularly vulnerable to contracting COVID-19 [201-204]. Moreover, several clinical studies reported that patients with diabetes were more likely to experience severe COVID-19 infection due to weak immune response, hyperinflammation, and underlying metabolic and cardiovascular issues [203]. COVID-19 infection cross-talks with several pathways discussed earlier that result in the worsening of disease conditions in diabetes as well as diabetic complications. Importantly, these pathways majorly include the inhibition of ACE-2 and Ang.1-7, which are responsible for alleviating inflammation and oxidative stress during diabetes. Consequently, it was found that the COVID-19 virus more preferentially binds to glycated hemoglobin, which is abundant in diabetic patients [205]. Besides, in hyperglycemic conditions, patients have a much smaller count of lymphocytic cells that are majorly involved in immune response [206]. However, due to the high levels of cytokines and inflammatory markers in diabetic-compromised patients, COVID-19 easily penetrates and replicates in the body and further worsens the disease condition, leading to an increased risk of morbidity and death.

COVID-19 has a complex relationship with CVD, particularly in diabetic patients, but specific pathophysiological mechanisms remain under investigation. Recent studies have identified elevated levels of troponin I and D-dimer, which are markers of cardiac injury and inflammation in COVID-19 patients with pre-existing CVD [133]. Furthermore, recombinant ACE2 drugs have shown promise in preclinical studies as potential inhibitors of viral entry, which may reduce cardiovascular complications [146].

Comprehensive management of high-risk groups, such as patients with hypertension, diabetes, or obesity, is essential. These patients, particularly older men and those with severe disease, should receive appropriate anticoagulation therapy and close monitoring. Continuing standard CVD treatments during COVID-19 is advised, with some evidence suggesting potential benefits from ACE inhibitors, statins, and lipid-lowering drugs, though their prophylactic use requires further study. Additionally, the interaction between diabetes and COVID-19 requires careful glucose management and preventive strategies to reduce disease severity and mortality. Long-term follow-up of severe COVID-19 survivors is crucial to understand potential cardiovascular complications. Future research must focus on the genetic predispositions, pathophysiological processes, and therapeutic interventions to better manage the dual burden of COVID-19 and CVD.

Neuropathy, as a complication of DM, also requires attention due to the varied manifestations of the virus, including its potential to cause debilitating and painful neuropathies. Alternative therapeutic approaches, such as nutraceuticals, have shown promise in relieving symptoms and could represent future research directions. The pandemic has also significantly impacted ophthalmology, with reports of conjunctivitis and other eye conditions linked to COVID-19. Managing these ocular manifestations and the potential retinopathies resulting from COVID-19 treatments will be areas of ongoing concern. Moreover, the sedentary lifestyle and poor diet resulting from pandemic restrictions may worsen diabetes control, increasing the risk of severe diabetic retinopathy. Healthcare professionals must be vigilant in monitoring and treating these complications, emphasizing the need for multidisciplinary approaches to manage the broad spectrum of COVID-19's impact.

As we continue to unravel the complex interplay between COVID-19 and DM, several key areas warrant further investigation to improve patient outcomes and therapeutic strategies. The ongoing research must focus on understanding the molecular mechanisms underlying the exacerbation of diabetic complications in COVID-19 patients. Specifically, elucidating the role of the ACE2 and DPP4 receptors in mediating viral entry and their subsequent impact on glycemic control is crucial. Additionally, exploring the implications of chronic inflammation and immune dysregulation in diabetic patients infected with SARS-CoV-2 can shed light on potential therapeutic targets to mitigate severe disease outcomes. One promising avenue for future research is the development of tailored pharmacological interventions that address the specific needs of diabetic patients with COVID-19. For instance, while current glucose-lowering agents such as DPP4 inhibitors have shown limited effectiveness in reducing COVID-19 vulnerability, their potential benefits in combination with other therapeutic agents should be explored. Investigating the efficacy and safety of SGLT2 inhibitors in this patient population is also imperative, given their contraindication in certain COVID-19 conditions. Moreover, the role of insulin therapy in acute glucose management during COVID-19 infection should be further elucidated to establish evidence-based guidelines for clinical practice.

In this perspective, the integration of precision medicine holds significant promise in enhancing the management of diabetic patients with COVID-19. By leveraging genomic, proteomic, and metabolomic data, researchers can identify biomarkers predictive of disease severity and therapeutic response. Such insights can facilitate the stratification of patients based on their risk profiles and guide personalized treatment regimens. Additionally, machine learning and artificial intelligence algorithms can be employed to analyze large datasets and uncover novel associations between patient characteristics, treatment modalities, and clinical outcomes. Further, longitudinal studies are essential to understand the long-term impact of COVID-19 on diabetic patients. Given the chronic nature of DM and the potential for persistent viral effects, it is important to monitor patients over extended periods to identify delayed complications and assess the durability of therapeutic interventions. These studies should also consider the influence of COVID-19 on the progression of diabetes-related comorbidities, such as cardiovascular disease, chronic kidney disease, and neuropathy. Understanding these dynamics can inform the development of comprehensive care models that address the complicated needs of this vulnerable population. Public health strategies must also be tailored to address the unique challenges faced by diabetic patients during the COVID-19 pandemic. Efforts to enhance vaccination coverage, promote preventive measures, and ensure timely access to healthcare services are critical. Moreover, targeted education campaigns can raise awareness about the importance of glycemic control and adherence to treatment regimens among diabetic patients. Collaborations between healthcare providers, policymakers, and patient advocacy groups can facilitate the dissemination of best practices and ensure that diabetic patients receive optimal care during the ongoing pandemic and beyond.

Exploring the socio-economic determinants of health in diabetic patients with COVID-19 is another critical area for future research. The pandemic has disproportionately affected marginalized communities, exacerbating existing health disparities. Investigating the impact of factors such as income, education, access to healthcare, and social support on disease outcomes can inform the development of equitable healthcare policies. Additionally, community-based interventions that address these determinants can enhance the resilience of vulnerable populations and improve their overall health outcomes. The potential for repurposing existing drugs for the treatment of COVID-19 in diabetic patients is an auspicious research direction. Given the urgency of the pandemic, identifying and validating the efficacy of readily available pharmacological agents can expedite the development of effective treatments. Clinical trials assessing the safety and efficacy of drugs such as metformin, GLP-1 receptor agonists, and other anti-diabetic medications in the context of COVID-19 can provide valuable insights into their therapeutic potential. Also, understanding the mechanisms by which these agents modulate immune responses and inflammatory pathways can reveal novel therapeutic targets.

Finally, international collaborations and data-sharing initiatives are essential to accelerate research progress and disseminate findings globally. The COVID-19 pandemic has highlighted the importance of coordinated efforts in addressing public health crises. Establishing platforms for the exchange of clinical data, research findings, and best practices can enhance the collective understanding of the interplay between COVID-19 and diabetes. Moreover, fostering partnerships between academic institutions, healthcare organizations, and industry stakeholders can facilitate the translation of research discoveries into clinical applications.

## CONCLUSION

The findings on COVID-19 and diabetes complications elucidate some clinical concerns, which means glycemic control is crucial in patients with diabetes, and strict observation of diabetic patients is required. Considering COVID-19, SARS-CoV-2 intensifies immune disorder and raises the danger of adverse outcomes in diabetic patients; thus, it is crucial to strictly adhere to the prescribed medications and continuously self-monitor blood glucose levels. Quarantine measures should be used to avoid infections and the resulting complications, but inevitable negative consequences. Healthcare providers should stress healthy living styles, including a balanced diet and exercise, to reduce the potential dangers of Global quarantine measures. Next, telehealth services should be embraced to minimize direct contact while maintaining treatment flow. The general vulnerability of diabetic patients in terms of worst-case COVID-19 consequences underlines the necessity of adopting an integrative approach to the problem. Hyperglycemia is a modifiable risk factor with a highly significant correlation with worsened disease outcomes, although the exact mechanisms through which hyperglycemia affects COVID-19 patients have yet to be explored comprehensively. Diabetes is among the comorbidities that have been found to exacerbate COVID-19 in patients, hence an association that requires appropriate public health measures to lower risks and enhance outcomes. Measures relevant to prevent and control the disease include early isolation, proper identification, and rapid management of the infection and its outcomes, thus improving prognosis.

Finally, it is found that ACE-2 receptor is involved with pancreatic β-cell and may be responsible for the effects of both Type I and Type II diabetes during COVID-19 infection. A distinct pattern of risk factors characterizes each type of diabetes, and therefore, it is crucial for future studies on diabetes to address genetic factors genes on pathophysiology and targeted individualized treatment. While expanding the understanding of this intricate connection between diabetes and COVID-19, new approaches for the effective erasure of the detrimental influence of the term COVID-19 on patients with diabetes worldwide must be sought.

## Figures and Tables

**Fig. (1):**
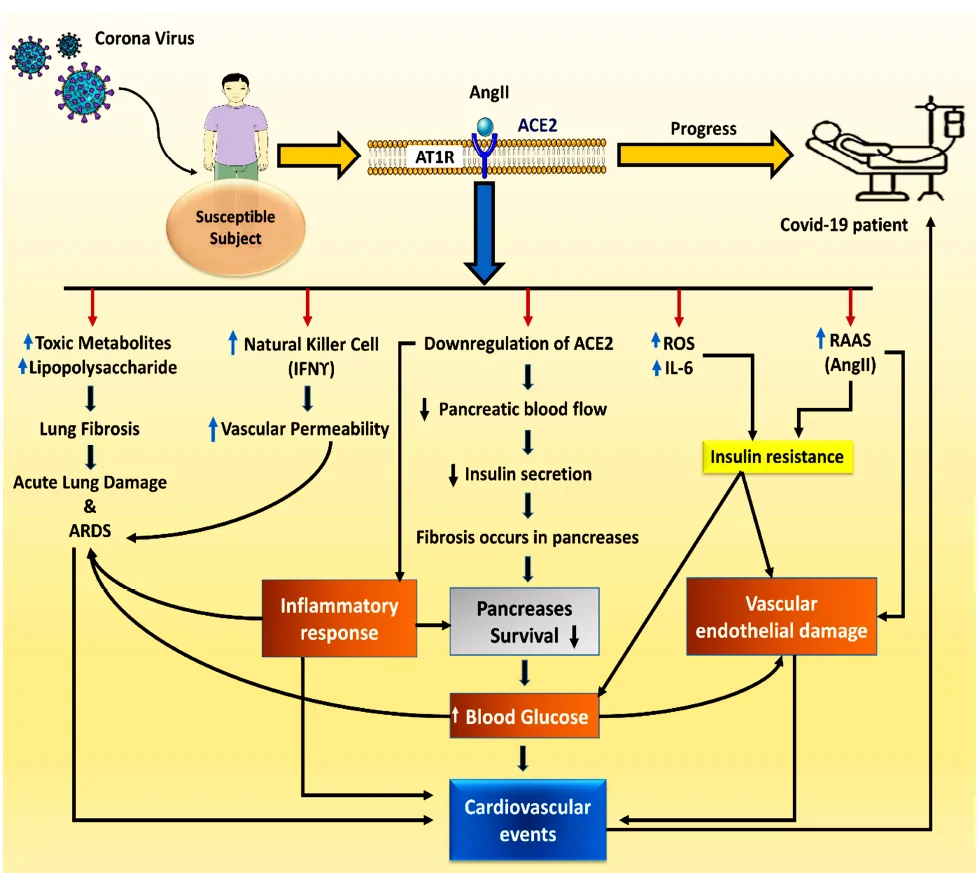
COVID-19 exacerbates diabetic complications.

**Fig. (2):**
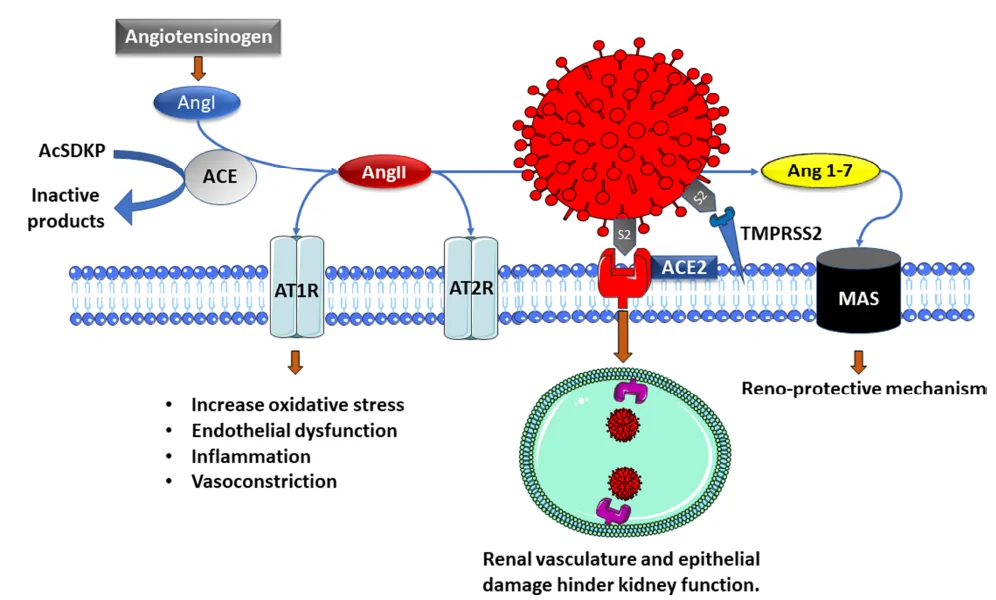
SARS-CoV-2 infection-associated disruption in the renin-angiotensin system causes kidney failure.

**Fig. (3):**
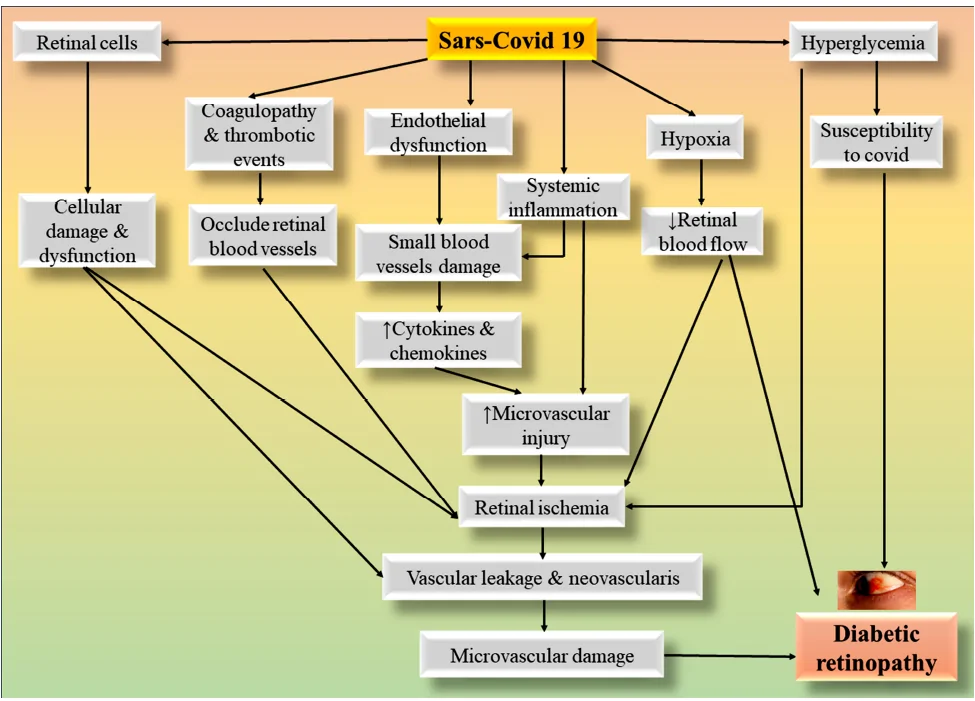
SARS-CoV-2 causes diabetic retinal complications.

**Fig. (4):**
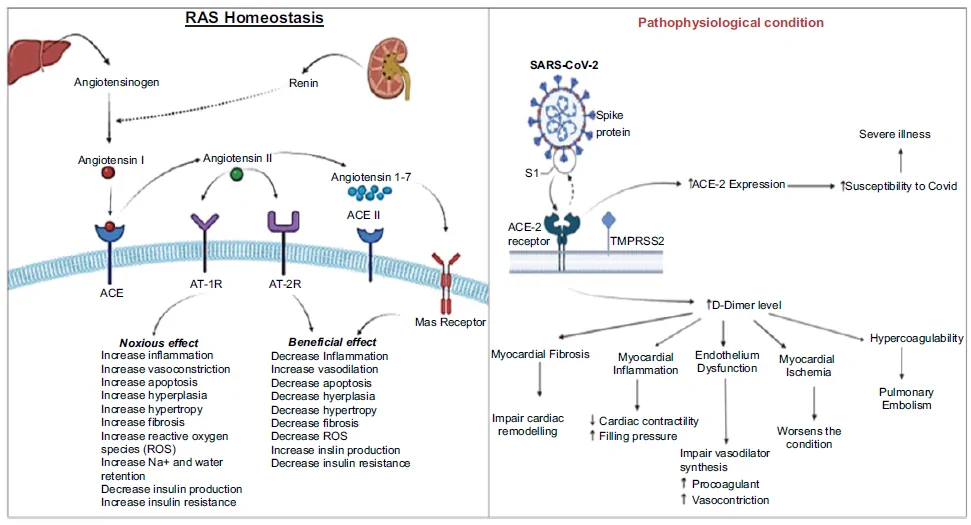
The possible pathogenesis of cardiovascular complications in COVID-19.

**Table 1:** Different variants of SARS-COV-2 virus.

**Sars-CoV 2 Variant (WHO Name)**	**Strain Name**	**Variant Name**	**Mutation Sites**	**Position of Amino Acid Mutation**	**1^st^ Reported Place**	**References**
Alpha	D614G	--	D614G	Aspartic acid (D) to glycine (G) at 614	China German	[44]
	B.1.1.7	VOC-2020122/01, or 201/501Y.V1	ΔH69/ΔV70 N501Y P681H Other-nucleocapsid (N) protein and ORF8	Deletion of histidine at 69 and valine at 70 asparagine (N) to tyrosine (Y) at Proline (P) to histidine (H) at 681	United Kingdom	[45, 46]
	B.1526	--	E484K S447N D253G D614G	Glutamic acid (E) to lysine (K) at 484 Serine (S) to asparagine (N) at 447 Aspartic acid (D) to glycine (G) at 253 Aspartic acid (D) to glycine (G) at 614	New York, USA	[47, 48]
Beta	B.1.351	501Y.V2	E484K N501Y K417N	Glutamic acid (E) to lysine (K) at 484 Sparagine (N) to tyrosine (Y) at 501 Lysine (K) to asparagine (N) at 417	South Africa	[47]
	B.1.1.28.1	--	E484K	Glutamic acid (E) to lysine (K) at 484	Brazil	[49]
Gamma	P.1	--	E484K N501Y K417T	Glutamic acid (E) to lysine (K) at 484 Asparagine (N) to tyrosine (Y) at 501 Lysine (K) to threonine (T) at 417	Brazil	[50]
Delta	B.1.617.2	--	L452R T478K P681R D614G	Leucine (L) to arginine (R) at 452 Threonine (T) to lysine (K) at 478 Proline (P) to arginine (R) at 681 Aspartic acid (D) to glycine (G) at 614	India	[51]
Delta plus	B.1.617.2.1	--	D614G K417N P861R T19R D950N T478K L452R ∆157, ∆158	Aspartate (D) to glycine (G) at 614 Lysine (K) to asparagine (N) at 417 Proline (P) to arginine (R) at 681 Threonine (T) to arginine (R) at 19 Aspartic acid (D) to asparagine (N) at 950 Threonine (T) to lysine (K) at 478 Leucine (L) to arginine (R) at 452 Deletion of amino acid at 157 & 158	United States	[51, 52]
Omicron	B.1.1.529	--	ΔH69/ΔV70 ΔY144 N501Y E484A Q493R T478K K417N	Deletion of Histidine at 69 and valine at 70 Deletion of a tyrosine amino acid at 144 Asparagine (N) to tyrosine (Y) at 501 Glutamic acid (E) to alanine (A) at 484 Glutamine (Q) to arginine (R) at 493 Threonine (T) to lysine (K) at 478 Lysine (K) to asparagine (N) at 417	South Africa	[53, 54]
Epilson	B.1427 B.1.429	20C/S:452R	L452R S13I W152C D614G T19R	Leucine (L) to arginine (R) at 452 Serine (S) to isoleucine (I) at 13 Tryptophan (W) to cysteine (C) at 152 Aspartic acid (D) to glycine (G) at 614 Threonine (T) to arginine (R) at 19	California (USA)	[55, 56]
Kappa	B.1.617.1	20A/S:154K	L452R E484Q T478K	Leucine (L) to arginine (R) at 452 Glutamic acid (E) to glutamine (Q) at 484 Threonine (T) to lysine (K) at 478	India	[57]
Iota	B.1.526	20C/S:484K	D80G T95I Y144 D253G L452R S477N E484K D614G A701V T859N D950H Q957R	Aspartic acid (D) to glycine (G) at 80 Threonine (T) to isoleucine (I) at 95 Deletion of tyrosine (Y) at 144 Aspartic acid (D) to glycine (G) at 253 Leucine (L) to arginine (R) at 452 Serine (S) to asparagine (N) at 477 Glutamic acid (E) to lysine (K) at 484 Aspartic acid (D) to glycine (G) at 614 alanine (A) to valine (V) at 701 Threonine (T) to asparagine (N) at 859 Aspartic acid (D) to histidine (H) at 950 Glutamine (Q) to arginine (R) at 957	New York	[57, 58]
Zeta	P.2	20J	E484K D614G V1176F	Glutamic acid (E) to lysine (K) at 484 Aspartic acid (D) to glycine (G) at 614 Valine (V) to phenylalanine (F) at 1176	Brazil	[59]
Eta	B.1.525	20A/S:484K	A67V 69del,70del,144del E484K D614G Q677H F888L	Alanine (A) to valine (V) at 67 Deletion of amino acid at 69, 70, 144 Glutamic acid (E) to lysine (K) at 484 Aspartic acid (D) to glycine (G) at 614 Glutamine (Q) to histidine (H) at 677 Phenylalanine (F) to leucine (L) at 888	United Kingdom, Nigeria	[60]
Theta	P.3	--	--	--	Philippines	[61, 62]
Lamda	C.37	--	Δ246-252 L452Q G75V T76I T859N D614G	Deletion of amino acid at 246 & 252 Leucine (L) to glutamine (Q) at 452 Glycine (G) to valine (V) at 75 Threonine (T) to isoleucine (I) at 76 Threonine (T) to asparagine (N) at 859 Aspartic acid (D) to glycine (G) at 614	Peru	[62]
Mu	B.1.621	--	E484K N501Y	Glutamic acid (E) to lysine (K) at 484 Asparagine (N) to tyrosine (Y) at 501	Columbia	[62]
R.1	-	--	W152L E484K G769V	Tryptophan (W) to leucine (L) at 152 Glutamic acid (E) to lysine (K) at 484 Glycine (G) to valine (V) at 769	Japan	[63]
B.1.621.1 (sublineage of Mu variant)	B.1.621.1	--	--	--	United States	[64]
B.1.640	B.1.640	--	--	--	France	[65]

**Table 2:** Possible cardiovascular ailments regarding COVID-19.

Manifestation	Description	Remarks	References
Acute cardiac injury	In human cells, SARS-CoV-2 enters and binds to ACE2, which alters the ACE2 signaling pathway and elevates the troponin I level.	The most frequent cardiovascular problem reported. Troponin I relate to inflammatory biomarkers; is an important correlation between CVD and inflammatory activity triggered by viral infection.	[28]
Systemic inflammation	Overproduction of inflammatory cytokines *i.e*; IL-6 and TNF-α.	More severe forms of COVID-19.	[9]
Altered myocardial demand	Alteration in supply and demand ratio of cardio-metabolites.	It can cause hypoxia and leads to acute respiratory illness and acute myocardial injury.	[143]
Adverse effects of various therapies	Various therapies which focus to treat COVID-19 but have noxious effect on cardiovascular system such as antiviral, medication, steroids, monoclonal antibody, oxygen therapy, thromboprophylaxis.	Causes cardiovascular complications, such as myocarditis, arrhythmias, and blood clot formation.	[144]
Myocardial inflammation	Due to COVID-19 cardiac contractility strength decreases and generating ionotropic deficit and filling pressure increases.	Causes acute heart failure.	[145]
Endothelium dysfunction	Vascular endothelium impairs synthesis of vasodilation and vasoconstriction and increase procoagulation.	Involve in pathophysiology of hypertension and atherosclerosis in insulin resistance condition.	[146]
Myocardial fibrosis	SARS-CoV-2 directly infect cardiac cells through the ACE2 receptor in heart, triggering an inflammatory response, leading to tissue damage and fibrosis.	Impair cardiac remodelling.	[147]

**Table 3:** Treatment of diabetic complications in the context of COVID-19.

**Diabetic Neuropathy**				
**Sr. No.**	**Class of Drugs/ Supplements**	**Examples**	**Mechanism of Action**	**References**
**1.**	GABA Analogs	Gabapentin, Pregabalin	Modulates calcium channels, reducing excitatory neurotransmitter release.	[85]
**2.**	SNRIs	Duloxetine, venalafaxine	Enhances serotonin and norepinephrine activity in the central nervous system.	[85]
**3.**	TCA	Amitryptiline, imipramine	Inhibits the reuptake of serotonin and norepinephrine.	[85]
**4.**	Opioid analgesic	Tramadol	Acts as a weak opioid agonist and inhibits the reuptake of serotonin and norepinephrine.	[85]
**5.**	Alkaloids	Capsaicin patches	Desensitizing nociceptive nerve endings, leading to the depletion of substance P, ultimately reducing pain transmission signals in diabetic neuropathy.	[85]
**6.**	Local Anesthetic	Lidocaine patches	Blocking voltage-gated sodium channels in peripheral nerves, thereby inhibiting the generation and conduction of pain signals.	[85]
**7.**	Neurotoxins	Botulinum toxin-A (BTX-A)	Inhibiting the release of acetylcholine at the neuromuscular junction, leading to muscle relaxation and subsequently reducing neuropathic pain.	[85]
**8.**	Supplements	α-lipoic acid	↑ Endogenous antioxidants (*e.g.*, GSH) ↓ Oxidized vit-C and E Cytokines, proinflammatory factors	[183-185]
**9.**	Supplements	Acetyl carnitine	↑ Expression of nerve growth factors and peripheral nerve regeneration ↑ Nerve fibers ↓Pain	[186, 187]
**10.**	Vitamins	Vitamin-D	↑ Expression of two antimicrobial peptides (cathelicidin & β-defennin) to improve innate immunity.	[188, 189]
**11.**	Vitamins	B1, B6 and B12	↓ ↓ Oxidative stress ↓ Nerve Conduction C-reactive protein Block nerve conduction and improve peripheral symptoms.	[190-193]
**Diabetic Retinopathy**				
**12.**	Injection	Anti-VEGF	Stops damage from the abnormal blood vessel and slow down vision loss.	[194]
**13.**	Antimalarial	Hydroxychloroquin	Lower viral load and HbA1c.	[83, 195]
**Diabetic Nephropathy**				
**14.**	DPP-4 inhibitor	Sitagliptin & linagliptin	Inhibit the virus entry into target cells and lower blood glucose level.	[196, 197]
**15.**	miRNAs treatment	miR-3154	Inhibits SARS-CoV-2 infection by targeting its protein-expressing genes.	[105]
**16.**	miRNAs treatment	miR-5197-3p	Inhibits SARS-CoV-2 by binding to the viral genome.	[105]
**17.**	miRNAs treatment	miR-1307-3p	Suppresses SARS-CoV-2 genome replication and regulates antiapoptotic protein.	[105]
**18.**	miRNAs treatment	miR-27b	Targets ACE2 receptors, potentially reducing SARS-CoV-2 infection.	[105]
**19.**	miRNAs treatment	miR-98-5p	Regulates TMPRSS2 levels, impacting SARS-CoV-2 infection.	[105]
**20.**	miRNAs treatment	miR-let-7	Targets TMPRSS2, reducing viral entry of SARS-CoV-2.	[105]
**21.**	miRNAs treatment	miR-4458	Inhibits SARS-CoV-2 by regulating TMPRSS2 levels.	[105]
**22.**	Glycoprotein hormone	Erythropoietin	Reversed NPR-1 expression, reduced proteinuria, and protected podocytes from AGE-mediated damage.	[198]
**Cardiovascular Complications**				
**23.**	ACEIs	Captopril	Lower angiotensin II levels, thereby reducing inflammation, fibrosis, and vascular permeability	[199]
**24.**	ARBs	Olmesartan	▪ Improve endothelial function, maintaining vascular health and preventing thrombosis. ▪ Reduced oxidative stress and inflammation.	[199]
**25.**	IL-6 receptor inhibitors	Tocilizumab	Reduce inflammation and improve insulin sensitivity.	[200, 201]
**26.**	Interleukin-1 receptor antagonists	Anakinra	↓ Cytokine storm and inflammation.	[178]

## LIST OF ABBREVIATIONS

DM: Diabetes Mellitus
ACE2: Angiotensin-converting Enzyme 2
T1DM: Type I Diabetes Mellitus
T2DM: Type II Diabetes Mellitus
SARS-CoV-2: Severe Acute Respiratory Syndrome Coronavirus 2
RAAS: Renin-angiotensin-aldosterone System
CKD: Chronic Kidney Disease
BUN: Blood Urea Nitrogen
GFR: Glomerular Filtration Rate
AKI: Acute Kidney Injury
ARDS: Acute Respiratory Distress Syndrome
CVD: Cardiovascular Disorder
